# Successful transcatheter mitral valve repair for mitral regurgitation complicating fulminant eosinophilic myocarditis: a case report

**DOI:** 10.1093/ehjcr/ytaf353

**Published:** 2025-07-25

**Authors:** Yudai Shiwaku, Tatsuya Aonuma, Yuya Kitani, Toshiharu Takeuchi, Naoki Nakagawa

**Affiliations:** Division of Cardiology and Nephrology, Department of Internal Medicine, Asahikawa Medical University, 2-1-1-1, Midorigaoka-higashi, Asahikawa 0788510, Japan; Division of Cardiology and Nephrology, Department of Internal Medicine, Asahikawa Medical University, 2-1-1-1, Midorigaoka-higashi, Asahikawa 0788510, Japan; Division of Cardiology and Nephrology, Department of Internal Medicine, Asahikawa Medical University, 2-1-1-1, Midorigaoka-higashi, Asahikawa 0788510, Japan; Division of Cardiology and Nephrology, Department of Internal Medicine, Asahikawa Medical University, 2-1-1-1, Midorigaoka-higashi, Asahikawa 0788510, Japan; Division of Cardiology and Nephrology, Department of Internal Medicine, Asahikawa Medical University, 2-1-1-1, Midorigaoka-higashi, Asahikawa 0788510, Japan

**Keywords:** Eosinophilic myocarditis, Fulminant myocarditis, Cardiogenic shock, Mitral regurgitation, Transcatheter mitral valve repair, Case report

## Abstract

**Background:**

Fulminant eosinophilic myocarditis (EM) has a poor prognosis. Acute severe mitral regurgitation (MR) is a life-threatening complication of EM: however, no established treatment thereof exists.

**Case summary:**

Herein, we report a case of a 70-year-old woman diagnosed with fulminant EM. She was in cardiogenic shock and treatment was initiated with an implanted intra-aortic balloon pump in addition to steroids and inotropes. Functional MR gradually worsened, and cardiogenic shock did not improve. Our cardiology team discussed the treatment plan and performed transcatheter mitral valve repair (TMVr). Immediately after the procedure, MR was well-controlled, and the patient’s hemodynamics improved dramatically. After discharge, there was no recurrence of heart failure.

**Discussion:**

We identified two important clinical issues: first, acute functional MR requiring invasive treatment can be associated with fulminant EM; and second, TMVr is useful as an invasive treatment strategy for such MR. Although MR caused by EM improves with pharmacological therapies in many cases, invasive treatment strategies may be required in some case, as seen in this case. Compared with surgeries, TMVr is less invasive and carries a lower risk of postoperative low cardiac output syndrome compared with surgical procedures when MR is well-controlled. If the response to pharmacological therapies is poor, early TMVr should be considered.

Learning points

- Mitral regurgitation (MR) is a complication of eosinophilic myocarditis (EM). MR can be fatal in fulminant cases because it may exacerbate haemodynamic instability and cause low cardiac output syndrome.
- No established treatment exists for MR secondary to EM. Although MR improves with immunosuppressive therapies alone in most reported cases, some cases may require invasive interventions. Among the invasive interventions, only surgeries have been reported thus far; however, transcatheter mitral valve repair may also be considered an effective option.

## Introduction

Eosinophilic myocarditis (EM) presents with a wide range of clinical manifestations, from asymptomatic to fulminant cases. In fulminant cases, low cardiac output syndrome (LCOS) and fatal arrhythmias due to the spread of inflammation to both ventricles often lead to cardiogenic shock.^1,2^ Mitral regurgitation (MR) is a common complication of EM. Despite a growing body of literature on EM and its treatment, only few reports discuss the use of surgeries as invasive interventions during the acute phase^3–5^ because MR improves with immunosuppressive therapies alone in most reported cases.^6–8^ Importantly, the use of transcatheter mitral valve repair (TMVr) has never been reported. TMVr is a procedure that repairs mitral valve dysfunction by transcatheter clipping of the anterior and posterior mitral valve leaflets using a clipping device. It is less invasive than surgery and may be effective for MR due to EM. Herein, we report a case of fulminant EM complicated by severe MR that was successfully treated with TMVr.

## Summary figure

**Figure:**
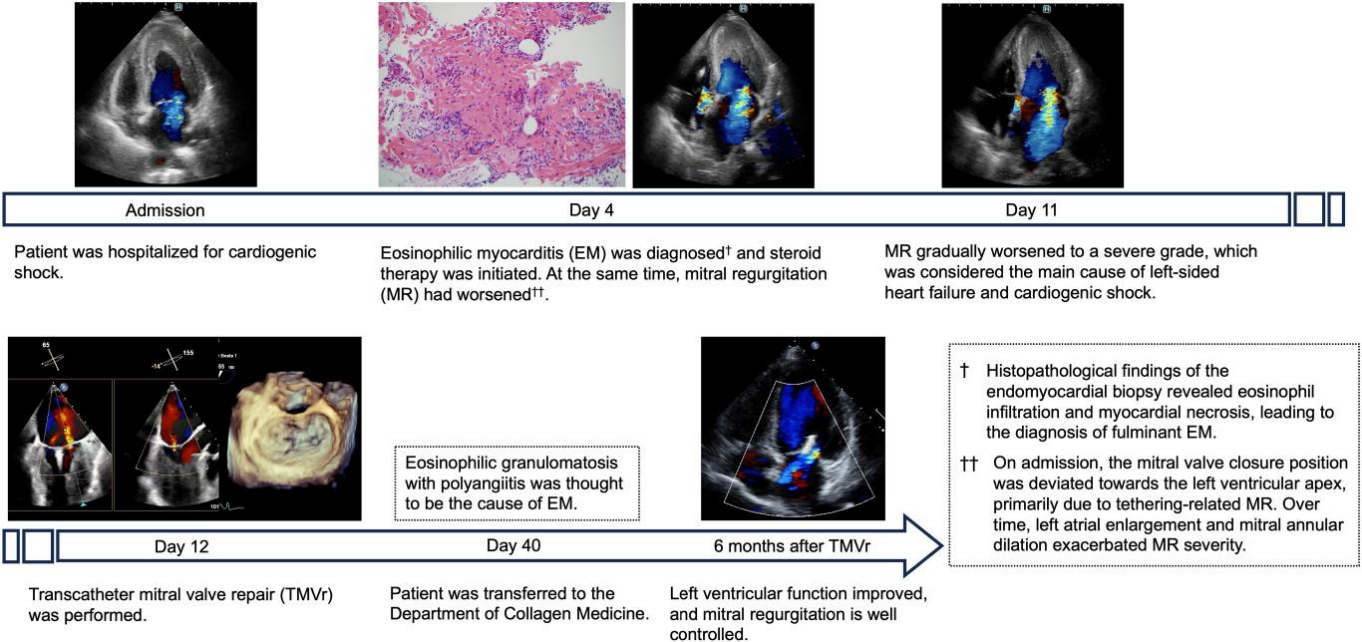


## Case presentation

**Figure 1:**
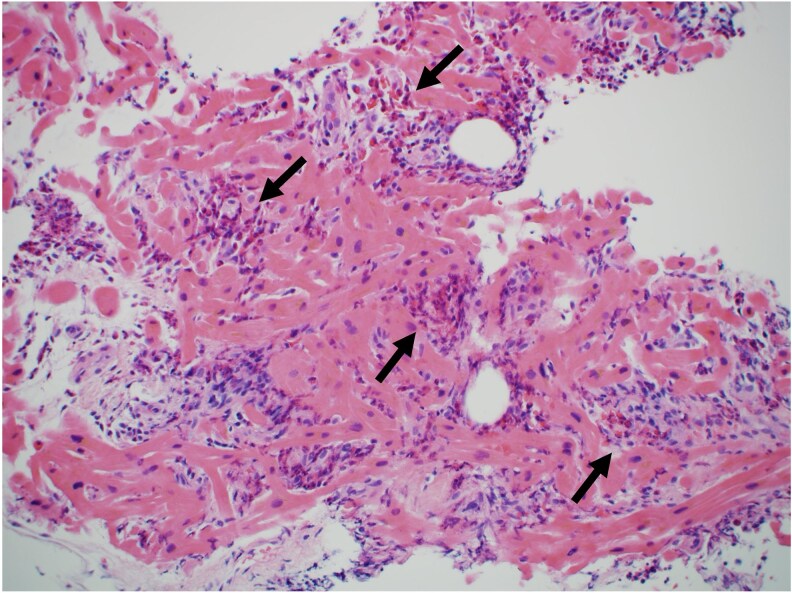
Endomyocardial biopsy. Inflammatory cell infiltration, predominantly consisting of eosinophils (black allow), and myocardial necrosis are observed (haematoxylin and eosin, original magnification: 200×).

**Figure 2:**
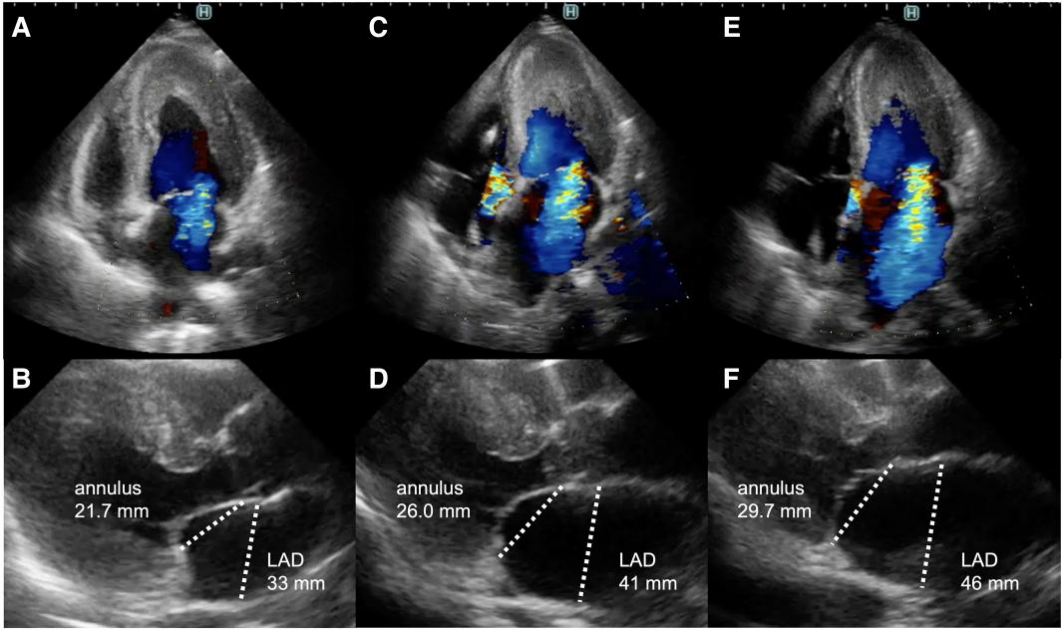
Transthoracic echocardiographic findings. On admission, the mitral valve closure position is seen to be dilated towards the LV apex, primarily due to tethering-related mild MR (*A* and *B*). The left atrium and annulus are enlarged, and the MR is worsened to moderate on day 4 (*C* and *D*). The MR is worsened to severe significantly on day 11 (*E* and *F*). LAD, left atrium dimension; LV, left ventricular; MR, mitral regurgitation.

**Figure 3:**
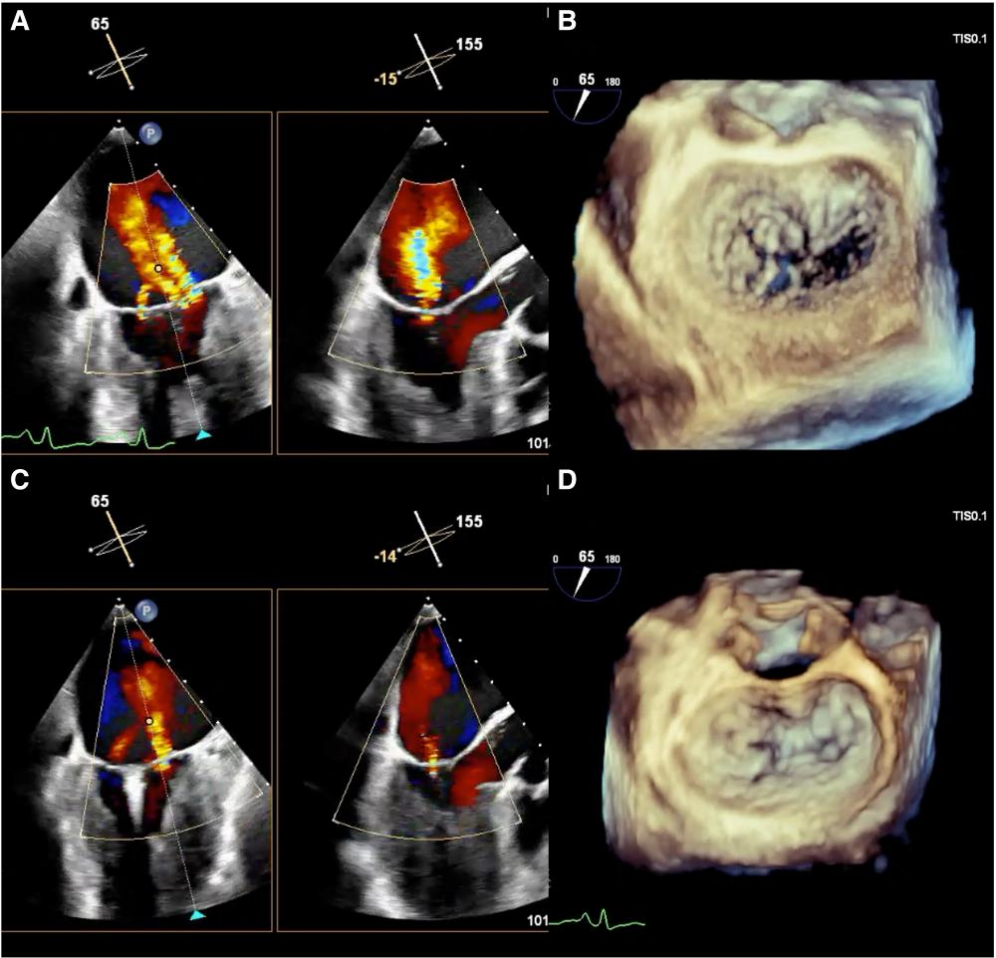
Transoesophageal echocardiographic findings during a transcatheter mitral valve repair procedure. An MR jet is identified in the A2-P2 lateral indentation and the valve leaflets are detached (*A* and *B*). NTW clip at A2-P2 centre is implanted due to thickening of the subvalvular myocardium at A2-P2 lateral (*C* and *D*). MR, mitral regurgitation.

**Figure 4:**
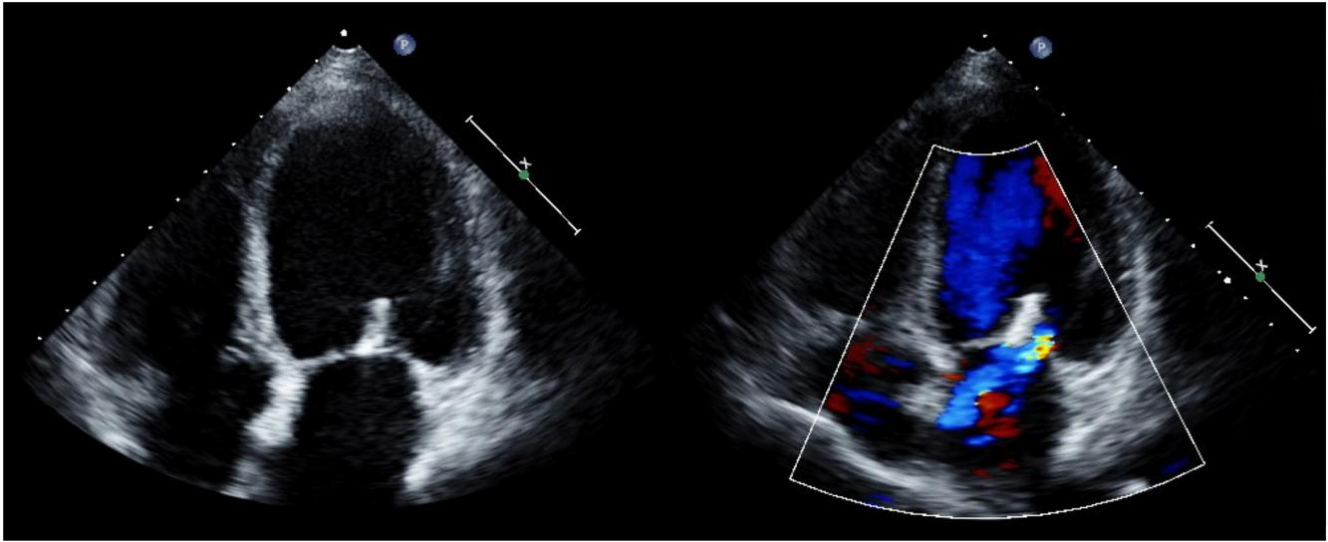
Transthoracic echocardiographic 6 months after TMVr findings. Left ventricular wall thickness and oedema have improved, and the MR is well-controlled.

A 70-year-old woman with bronchial asthma and chronic sinusitis presented to our hospital with generalized fatigue and dyspnoea. She had experienced worsening numbness and muscle weakness in both lower limbs for ∼1 month before admission. On admission, she exhibited jugular venous distension, cold extremities, and bilateral coarse crackles. An examination of the vital signs revealed a blood pressure of 92/70 mmHg, a heart rate of 148 beats/min with regular rhythm, a respiratory rate of 20 breaths/min, and an oxygen saturation of 91% on room air. Chest radiography revealed bilateral pulmonary oedema without an enlargement of the heart (cardiothoracic ratio 45%). Electrocardiography revealed atrial fibrillation, ST- segment depression in leads V4-6, and an incomplete left bundle branch block. Transthoracic echocardiography (TTE) demonstrated diffuse oedematous wall thickening in the left ventricle (LV) and a reduced LV cavity (end-systolic diameter of 29 mm and end-diastolic diameter of 38 mm). A reduced LV ejection fraction of 42% and an LV outflow tract velocity time integral of 8.3 cm indicated LCOS. At this time, no moderate or severe MR findings were observed. Blood test revealed elevated cardiac enzymes (troponin I 50 381 pg/mL; normal < 26.2 pg/mL, creatine kinase 413 IU/L; normal: 41–153 IU/L, and creatine kinase Muscle/Brain 24 IU/L; < 12 IU/L), eosinophilia (10 970 cells/μL; normal < 500 cells/μL), and elevation of IgE (1105 IU/mL; normal < 232 IU/mL). Given the strong suspicion of EM, we performed an urgent cardiac catheterisation and right ventricular endomyocardial biopsy (EMB). Coronary angiography revealed no noticeable stenosis of the coronary arteries. Right heart catheterisation revealed a Forrester subset IV classification, characterized by an elevated mean pulmonary capillary wedge pressure of 23 mmHg and a low cardiac index of 1.9 L/min/m^2^, consistent with cardiogenic shock. The patient was classified as SCAI Shock Stage C with preserved right heart function. Given the thickened left ventricular wall and reduced chamber size, the use of percutaneous ventricular assist device posed risks of inadequate drainage and haemolysis. Therefore, we introduced an IABP and initiated inotropes in the intensive care unit. Because EM due to eosinophilic granulomatosis with polyangiitis (EGPA) was strongly suspected, steroid pulse therapy (methylprednisolone 1000 mg/day for 3 days) was initiated, followed by prednisolone starting at 50 mg on day 4 of hospitalisation. Although the addition of intravenous cyclophosphamide (IVCY) was desirable given the severity of myocarditis, concerns regarding immunosuppression and bone marrow suppression led to the decision to introduce IVCY after the resolution of cardiogenic shock. On day 3, non-invasive positive pressure ventilation (NPPV) initiated because cardiogenic pulmonary oedema had not improved. On day 4, histopathological examination of the EMB revealed myocardial necrosis with infiltration of eosinophils and lymphocytes, leading to a diagnosis of fulminant EM (*Figure 1*). Follow-up TTE revealed a gradual reduction in LV wall thickening and improved cardiogenic shock; therefore, the IABP was weaned off on day 10. However, pulmonary oedema persisted, and the patient was difficult to wean from inotropes and NPPV. MR gradually worsened to a severe grade on day 11, which was considered the main cause of the left-sided heart failure and cardiogenic shock. On admission, the mitral valve closure position was deviated towards the LV apex, primarily due to tethering-related MR. Over time, left atrial enlargement and mitral annular dilation exacerbated MR severity (*Figure 2*). Based on our discussion with the heart team, TMVr was performed using the MitraClip G4 system (Abbott Vascular, Santa Clara, CA, USA) on day 12. Intraoperative transoesophageal echocardiography demonstrated an MR jet confirmed at A2-P2 lateral-lateral indentation. Because of the thickening of the subvalvular myocardium at A2-P2 lateral, we implanted NTW (wide and short clip arm) clip at A2-P2 centre (*Figure 3*). MR was controlled postoperatively, and subsequent right heart catheterisation revealed improved hemodynamics with a mean pulmonary capillary wedge pressure (11 mmHg) and a cardiac index (3.7 L/min/m^2^). The inotropes and NPPV were discontinued on day 13, and the patient was transferred to the general ward on day 20. Beta-blockers, mineralocorticoid receptor antagonists, and sodium-glucose cotransporter-2 inhibitors were introduced as guideline-directed medical therapy (GDMT) for heart failure. On day 40, the patient was transferred to the Department of Collagen Medicine for the initiation of IVCY for EGPA. IVCY was administered six times over the course of 1 year, followed by mepolizumab. Prednisolone was gradually tapered and discontinued ∼1 and 1.5 years. TTE performed 6 months after TMVr demonstrated normalized LV function and mild residual MR (*Figure 4*). Cardiac MRI with T2 mapping performed around the same time revealed no myocardial oedema suggestive of ongoing inflammation. Gadolinium contrast was not administered due to concerns about allergic reactions associated with EGPA. After discharge, GDMT has been continued after discharge, and the patient remained clinically stable.

## Discussion

This report presents the case of a 70-year-old woman with fulminant EM complicated by severe MR, successfully managed with TMVr after failure of pharmacological therapies. The patient’s course provided two important clinical suggestions. First, acute functional MR requiring invasive strategy can be associated with fulminant EM. EM can manifest from being asymptomatic to resulting in cardiogenic shock and cardiac arrest.^1,2^ Severe ventricular systolic and diastolic dysfunction or fatal arrhythmias due to the spread of inflammation to both ventricles are the main factors affecting disease severity. In addition, several previous reports have reported severe MR secondary to EM and discussed the underlying mechanisms, including (i) tethering due to inflammation spreading to the LV, (ii) restricted mobility of the leaflets and chordae tendineae due to inflammation and thrombus spreading to the mitral valve complex, and (iii) detachment of the mitral valve leaflet associated with annulus dilation due to MR.^3–9^ In fulminant cases, MR resulting from the aforementioned pathologies can be fatal, because it may exacerbate haemodynamic instability. Although no established treatment is available for EM complicated by MR, immunosuppressive therapies have improved MR in many cases.^6–8^ Invasive treatment strategies have been chosen for chronic cases, such as Loeffler endocarditis, but have rarely been performed in fulminant cases.^3–5^ In fulminant cases with a poor initial response to pharmacological therapies, one option is to extend mechanical circulatory support (MCS) while waiting for an improvement in MR. However, considering the complications associated with MCS, choosing early invasive treatment may be a better option.

Second, TMVr proved useful in managing MR due to fulminant EM although it was performed as a bridge to recovery until shock resolution and myocarditis improvement in this case. Although several cases of surgical interventions for managing MR have been reported, this is the first report of treatment with TMVr. Surgeries performed during active myocarditis increase the risk of postoperative LCOS. In addition, surgical procedures have a high risk of postoperative thrombosis because EM occurs in a prothrombotic state owing to chemical mediators secreted from eosinophils, and intraoperative endocardial injury and an extracorporeal circuit may further increase thrombogenicity.^9^ Additionally, perioperative deaths due to thrombotic valves have been reported.^5^ Therefore, in most cases of mitral valve surgery, valve replacement using biological valves or valvuloplasty is selected. However, the risks of prosthetic valve dysfunction and thromboembolic events remain high. Compared with surgeries, TMVr is less invasive and carries a lower risk of postoperative LCOS when MR is well-controlled. Therefore, TMVr can be considered the first choice in cases requiring an invasive strategy for MR, although it also has a risk of thrombosis. If TMVr is ineffective in controlling MR, surgery can still be considered as an alternative treatment option, even after the TMVr. Generally, thrombosis has been reported in TMVr owing to damage to the endocardium and mitral valve complex during the procedure and left atrial pressure postoperatively.^10^ Additionally, EM may further promote thrombus formation, making postoperative thrombus assessment and anticoagulant therapy essential. In this case, perioperative anticoagulation with unfractionated heparin was administered, and no thrombus was observed during follow-up.

In conclusion, fulminant EM can cause acute functional severe MR, and TMVr is useful for managing this condition. If patients respond poorly to pharmacological treatment, early TMVr must be considered.

## Lead author biography


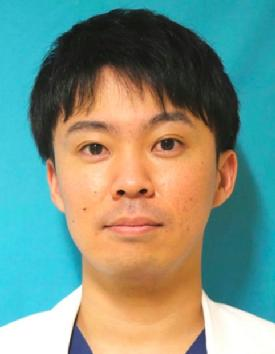


Dr. Yudai Shiwaku graduated in Asahikawa Medical University in 2017 and is currently working at Asahikawa Medical University Hospital as a cardiologist. He has a particular interest in valvular diseases and cardiogenic shock.

## Data Availability

The data in this article will be shared upon reasonable request.

## Notes

**Consent:** The authors confirm that written consent has been obtained from the patient, in line with the COPE guidelines, for the submission of this article.

**Funding:** None declared.
