# Correction to: Successful transcatheter mitral valve repair for mitral regurgitation complicating fulminant eosinophilic myocarditis: a case report

**DOI:** 10.1093/ehjcr/ytaf492

**Published:** 2025-11-22

This is a correction to: Yudai Shiwaku, Tatsuya Aonuma, Yuya Kitani, Toshiharu Takeuchi, Naoki Nakagawa, Successful transcatheter mitral valve repair for mitral regurgitation complicating fulminant eosinophilic myocarditis: a case report, *European Heart Journal - Case Reports*, Volume 9, Issue 8, August 2025, https://doi.org/10.1093/ehjcr/ytaf353

In the originally published version of the manuscript the first and last names of the fourth co-author were erroneously transposed. These have been emended in the article now to read: “Naoki Nakagawa”.

